# Effects of Tai Chi and brisk walking on the bone mineral density of perimenopausal women: A randomized controlled trial

**DOI:** 10.3389/fpubh.2022.948890

**Published:** 2022-08-22

**Authors:** Liang Cheng, Shuwan Chang, Benxiang He, Yang Yan

**Affiliations:** ^1^School of Sports Medicine and Health, Chengdu Sport University, Chengdu, China; ^2^Department of Humanities and Society of Sport, Sichuan Sports College, Chengdu, China

**Keywords:** Tai Chi, brisk walking, perimenopausal, female, exercise cessation, bone mineral density

## Abstract

**Background:**

There is a lack of information on whether the positive effect of Tai Chi (TC) and brisk walking (BW) exercise on bone mineral density (BMD) in perimenopausal women remains after exercise cessation. To compare the effects of regular TC and BW exercise on BMD in perimenopausal women and to analyze the maintenance effects after exercise cessation.

**Methods:**

The TC and BW groups performed 48 weeks of exercise. The BMD of the lumbar spine and dominant-side proximal femur was measured in all subjects at weeks 0, 48, 52, and 56.

**Results:**

Compared with baseline, the BMD of the lumbar spine (L_2−4_) at week 48 increased by 5.05% (*P* = 0.031) in the TC group, and the BMD of the femoral neck at week 48 increased by 8.23% (*P* = 0.031) in the BW group. At 4 and 8 weeks after exercise cessation, the BMD of L_2−4_ in the TC group was still increased by 5.05% (*P* = 0.041) and 5.05% (*P* = 0.023), respectively, and the BMD of the femoral neck was still increased by 8.23% (*P* = 0.018) and 9.41% (*P* = 0.007), respectively, in the BW group.

**Conclusion:**

Forty-eight weeks of TC exercise significantly increases the BMD of L_2−4_ in perimenopausal women, and BW exercise significantly increases the BMD of the femoral neck; these effects are maintained at 4 and 8 weeks after exercise cessation. These results suggest that these two exercises reduce the BMD decrease caused by aging.

## Introduction

With the increase in age, women experience degeneration of ovarian function, decreased estrogen secretion, and imbalance of the pituitary-ovarian axis, which leads to endocrine and autonomic disorders that cause perimenopausal syndrome (1). Perimenopausal women also commonly experience a decrease in bone mineral density (BMD) and even osteoporosis (2, 3). Therefore, there is a need to improve the BMD of perimenopausal women. Studies have confirmed that human BMD can be improved by appropriate exercise, such as regular resistance training (4, 5) and aerobic exercise (6, 7). Exercise causes the contraction of muscle groups and resulting stresses on the bones, inducing hormonal changes that affect bone metabolism (8, 9).

Aerobic exercise is preferred over resistance training by the middle-aged and older adult populations, as resistance training is considered difficult because it requires the participants to use devices or perform collective activities (10–12). However, the effect of aerobic exercise on human BMD is controversial. A meta-analysis analyzed the effect of aerobic exercise on BMD in middle-aged and older adults and concluded that an exercise duration of 24 weeks is not enough to increase the BMD, while a duration of more than 48 weeks significantly increases the BMD of the lumbar spine and greater trochanter (10). Tai Chi (TC) and brisk walking (BW) as the common aerobic exercise methods in the elderly, the exercise intensity is moderate. Previous studies found that the average energy expenditure in TC and BW practices were 3.1METs (13) and 3.6METs (4.8 km/h) (14). According to the Centers for Disease Control and Prevention, below 3METs are low intensity exercise, 3–6 METs are moderate intensity exercise, and >6 METs are high intensity exercise (15). It shows that TC and BW exercises are moderate-intensity exercises. Previous studies by our team have reported that 48 weeks of TC increases the BMD of the lumbar spine and femoral neck in older women, with TC achieving a better effect on the BMD than BW and square dance exercise (11).

It is also important to investigate how human BMD changes after exercise cessation. In one study, healthy older women who performed either TC or BW exercise for 16 weeks showed improvements in calcaneal BMD and bone metabolism parameters, and the BW group had better maintenance of calcaneal BMD at 8 weeks after exercise cessation than the TC group (16). Another study reported that older women who performed 24 weeks of whole-body vibration training still had a greater BMD of the greater trochanter than the control group at 8 weeks after exercise cessation (17). Although the positive effects of regular exercise are widely accepted, not everyone can persist with exercise over the long-term. Therefore, there is a need to investigate the maintenance of BMD after the cessation of regular exercise, as this information will affect the design of scientific research and effective exercise prescriptions (18). However, there is a lack of information on whether the positive effect of exercise on BMD in perimenopausal women remains after exercise cessation.

The purpose of the present study was to investigate the effects of TC and BW on the BMD of the lumbar spine and femur in perimenopausal women, and evaluate the maintenance effect after exercise cessation. The study hypothesis was that regular TC and BW exercises have a positive effect on the BMD of perimenopausal women, and that this effect is maintained after exercise cessation.

## Materials and methods

### Participants

Perimenopausal women were recruited from large community activity centers and parks around our university *via* interviews and posted advertisements. The inclusion criteria were: (1) age 45–55 years; (2) Kupperman index < 30; (3) hot flashes, sweating, irritability, and other menopausal symptoms; (4) no special exercise hobbies; (5) good health status as assessed *via* a general health examination; (6) provision of written informed consent. The exclusion criteria were: (1) dyskinesia; (2) current smoker or alcohol drinker; (3) intake of estrogen, calcium, vitamin D, anti-osteoporosis medication, and other drugs in the past 2 years; (4) osteoporosis diagnosed based on a *T* < −2.5 SD.

**Figure 1:**
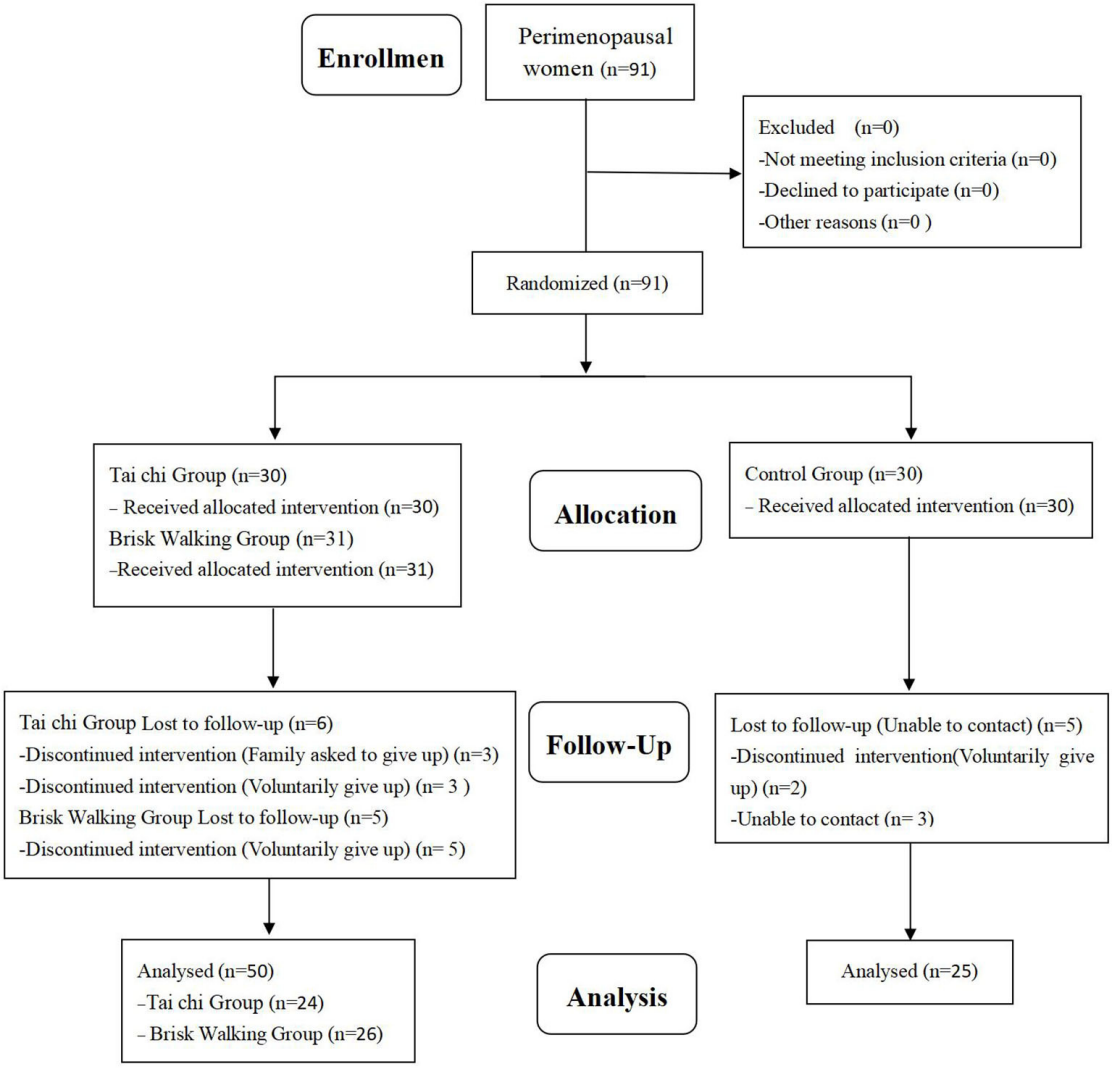
Participant selection flow diagram.

Referring to the results of previous studies on the effects of TC and BW on the BMD of humans (11, 12), the present study comprised a 3 (number of groups) × 4 (number of measurements) experimental design, with a predicted sample loss rate of approximately 15%. Using G-power software with the effect size set at 0.3, power set at 0.8, and α set at 0.05, the calculated required sample size was at least 90 subjects. Ninety-one perimenopausal women were initially recruited, and 16 withdrew before the end of the study period for personal or family reasons, giving a sample attrition rate of 17.5%. A final total of 75 subjects completed the entire experimental process. The patients were randomly divided into the TC group (*n* = 24), BW group (*n* = 26), and control group (*n* = 25) (Figure 1). There were no significant intergroup differences in age, height, and weight. Based on the Kupperman index, the degree of perimenopausal syndrome was mild in seven and moderate in two subjects in the TC group, mild in six and moderate in two subjects in the BW group, and mild in five and moderate in three subjects in the control group. The basic information of the three groups is shown in Table 1. This study was approved by the Ethics Committee of our university (approval no. SSCC02).

**Table 1:** Participants' basic information.

**Group**	**n**	**Postmenopausal/** **premenopausal/** **menopausal disorder**	**Age (years)**	**Height (cm)**	**Weight (kg)**	**Exercise time (weeks)**
Tai Chi group	24	6/12/6	50.2 ± 3.1	158.2 ± 7.1	59.3 ± 6.2	48
Brisk walking group	26	7/14/5	49.7 ± 3.7	157.6 ± 6.5	58.8 ± 7.0	48
Control group	25	6/13/6	50.1 ± 2.9	159.0 ± 5.9	59.2 ± 6.7	0

### Tai Chi and brisk walking interventions

Under the guidance of laboratory personnel, the TC and BW groups performed 60 min sessions of exercise (including 10 min of warm-up and relaxation) three times a week for 48 weeks. The exercise sessions were held from 07:00 to 08:00 am on Monday, Wednesday, and Friday in a park near our university. A 24-style simplified form of TC was selected for the TC group. The TC exercises have a fixed voice prompt, including 24 movements (~5 min is consumed to complete the exercise), and each movement completion time is fixed, for which the exercise rate is fixed. The intensity of the exercise can be determined in terms of how much the subject's center of gravity drops and the extent of the limb stretch. And the cadence of the BW group was not <90 steps/minute (4.8 km/h). The exercise intensity was based on that used in a previous study (11). Immediately after each exercise session, the subjects all counted their heart rate (using the pulse of the radial artery at the wrist) for 30 s as timed by a researcher with a stopwatch. The heart rate was controlled at 55–65% of the maximum heart rate (220–age), and the exercise intensity of subjects whose heart rate was above or below this range was appropriately decreased or increased at the next exercise session. In the weekly TC and BW groups, the total training duration was 180 min, and the practice intensity was shown as moderate intensity according to the heart rate. The control group maintained their original living habits and did not perform regular exercise. In accordance with the methods used in previous studies (18, 19). The control group was brought together thrice a week (1 h each time) to discuss knowledge related to BMD and exercise, or to discuss topics of interest to the subjects. The researchers conducted telephone interviews every week to record the living conditions of the TC, BW, and control groups, including the duration of exposure to sunshine, dietary habits, performance of additional physical exercise, and intake of drugs that affect the BMD. This was to ensure that the subjects did not perform any other form of physical exercise or took medications that affect the BMD during the study period. Subjects who did not meet the requirements were not included in the final analysis.

All subjects underwent BMD testing a total of four times: at week 0, week 48, week 52 (4 weeks after exercise cessation), and week 56 (8 weeks after exercise cessation).

### Bone mineral density testing

A Norland XR-46 dual-energy X-ray system (Norland, Fort Atkinson, WI, USA) was used to test the BMD of the lumbar spine and the proximal femur of the dominant side in the three groups. Five consecutive non-reentrant scans were performed at each of four measurement sites: the lumbar spine (L_2−4_), femoral neck, greater trochanter, and Ward's triangle; the measurement unit was g/cm^2^. The error coefficients were 1–2% for the lumbar spine, femoral neck, and greater trochanter, and 2.5–5% for Ward's triangle (11). The coefficient of variation in the lumbar vertebrae and the proximal femur was 1.1 and 1.85%, respectively (12).

### Data analysis

SPSS 17.0 software was used to calculate the mean ± standard deviation of the BMD at the four measurement sites in each of the three groups. Two-way ANOVA with a mixed design was used to test for group effects, time effects, and to determine whether there was an interaction between group (3) and time (4). If there was an interaction between group and time, one-way ANOVA with repeated measures was used to analyze the differences in the BMD at different timepoints within a group. If there was a main effect of time, *post-hoc* comparisons of the differences in the BMD across timepoints were performed. If there was a main effect of group, the difference in the BMD at the same timepoint in different groups was compared in a *post-hoc* manner. Bonferrone adjustment was used for *post-hoc* comparisons to ensure that the overall Type I error rate for each ANOVA was not >0.05. The significance level was set as α = 0.05.

## Results

**Figure 2:**
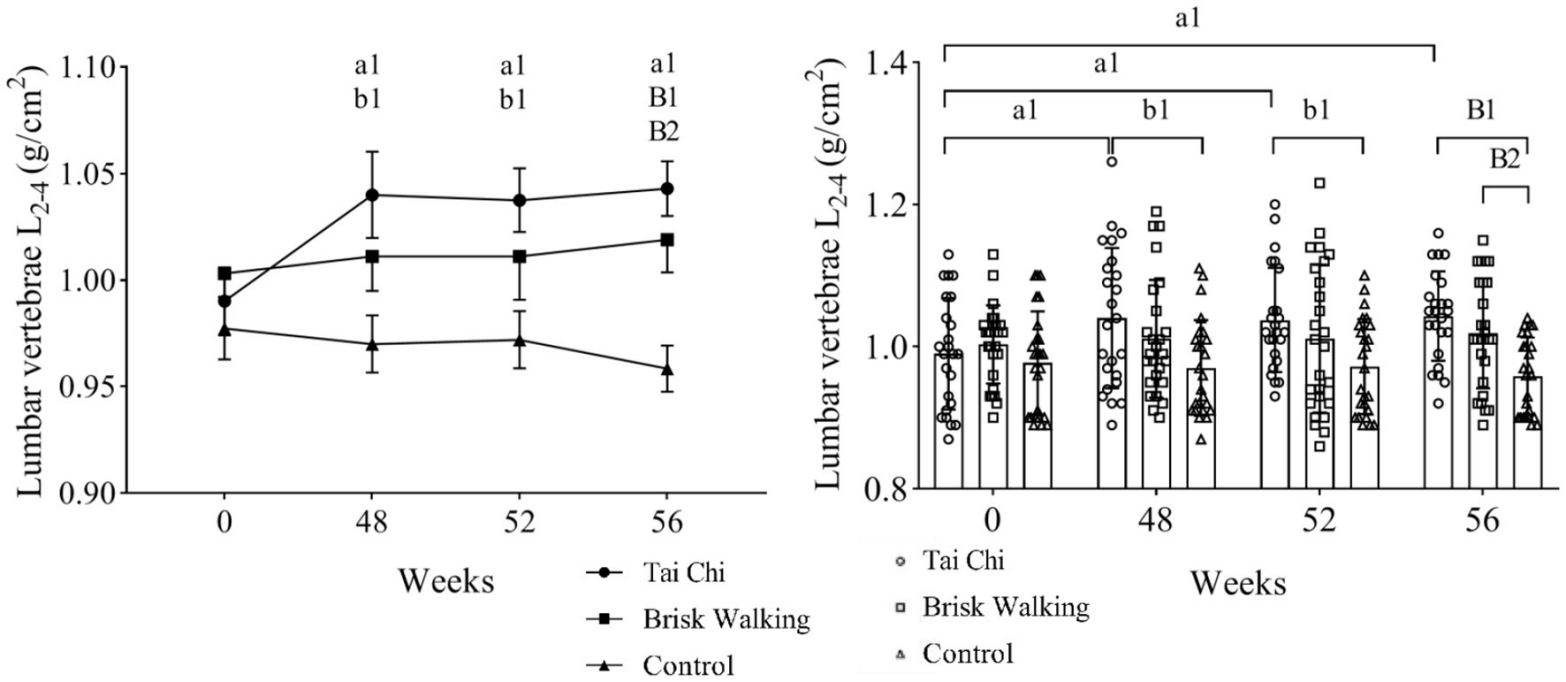
Bone mineral density of lumbar vertebrae L_2−4_. Fifty-two weeks is 4 weeks after exercise cessation, and 56 weeks is 8 weeks after exercise cessation. Tai Chi group intra-group comparison, in the comparisons of week 48, week 52, week 56 vs. week 0, a1 indicate differences of *P* < 0.05. Tai Chi group compared with the control group at the same timepoint, b1 indicate differences of *P* < 0.05, while B1 indicate differences of *P* < 0.01. Brisk Walking group compared with the control group at the same timepoint, B2 indicate differences of *P* < 0.01.

**Figure 3:**
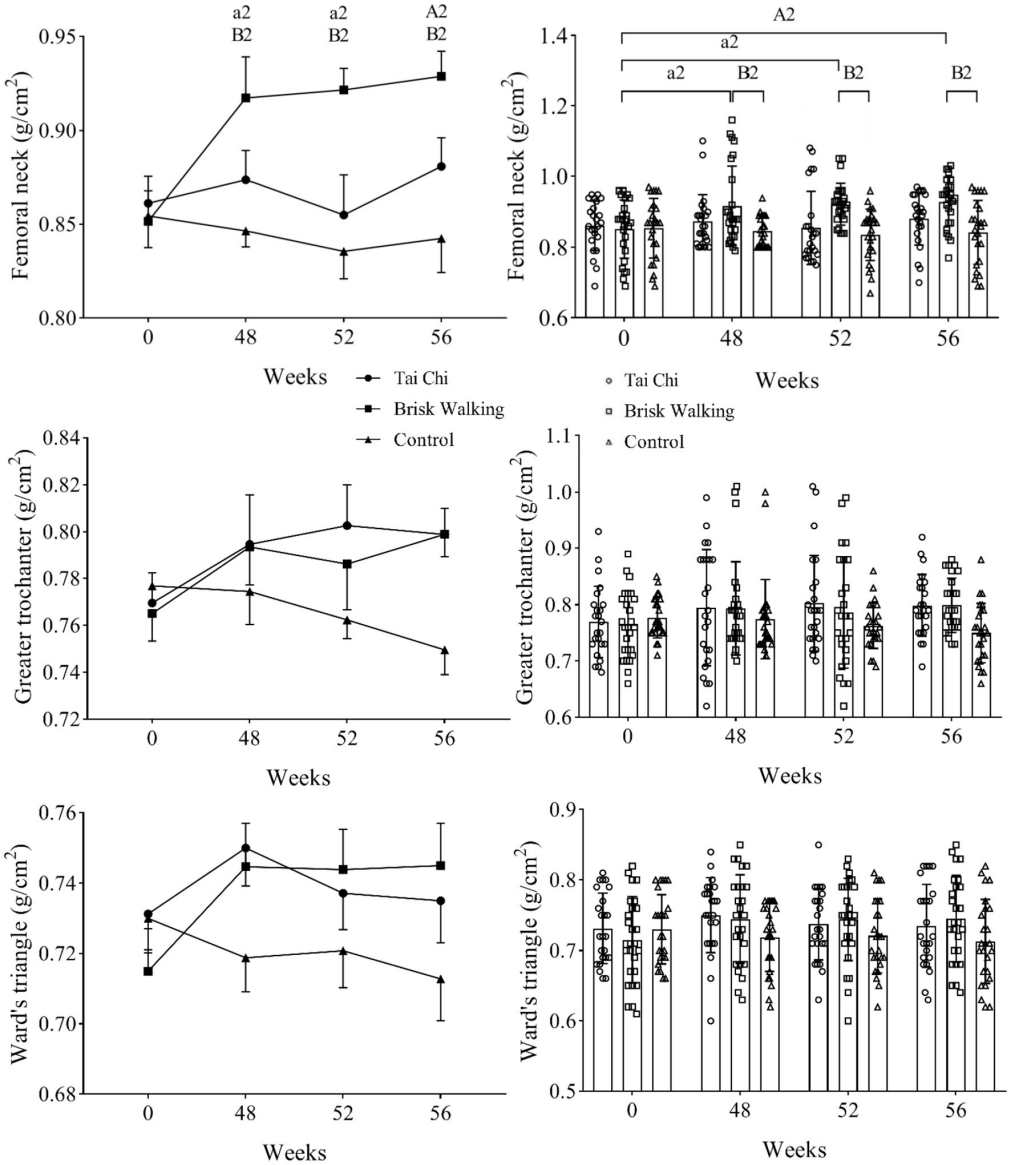
Bone mineral density of the proximal femur. Fifty-two weeks is 4 weeks after exercise cessation, and 56 weeks is 8 weeks after exercise cessation. Brisk Walking group intra-group comparison, in the comparisons of week 48, week 52, week 56 vs. week 0, a2 indicate differences of *P* < 0.05, while A2 indicate differences of *P* < 0.01. Brisk Walking group compared with the control group at the same timepoint, B2 indicate differences of *P* < 0.01.

The BMD results of the TC, BW, and control groups at different timepoints are shown in Figures 2, 3. Two-way ANOVA with a mixed design showed no interaction between group and time regarding the BMD of the lumbar spine [*F*_(6, 288)_ = 1.123, *P* = 0.349], femoral neck [*F*_(6, 288)_ = 1.978, *P* = 0.069], greater trochanter [*F*_(6, 288)_ = 1.133, *P* = 0.343], and Ward's triangle [*F*_(6, 288)_ = 1.026, *P* = 0.408].

The baseline BMD data at week 0 did not significantly differ between the TC (Kupperman index, mild and moderate, *n* = 9), BW (Kupperman index, mild and moderate, n = 8), and control groups (Kupperman index, mild and moderate, *n* = 8) [lumbar spine: *F*_(2, 22)_ = 0.811, *P* = 0.433; femoral neck: *F*_(2, 22)_ = 0.125, *P* = 0.918; greater trochanter: *F*_(2, 22)_ = 0.365, *P* = 0.711; Ward's triangle: *F*_(2, 22)_ = 0.628, *P* = 0.501], indicating that the samples were homogeneous. The baseline BMD data at week 0 did not significantly differ between the TC, BW, and control groups [lumbar spine: *F*_(2, 72)_ = 0.899, *P* = 0.412; femoral neck: *F*_(2, 72)_ = 0.097, *P* = 0.907; greater trochanter: *F*_(2, 72)_ = 0.307, *P* = 0.736; Ward's triangle: *F*_(2, 72)_ = 0.715, *P* = 0.493], indicating that the samples were homogeneous.

Compared with baseline, the BMD of the lumbar spine in the TC group at week 48 was significantly increased by 5.05% (*P* = 0.031, 95% CI: 0.0045–0.0955), and the BMD of the femoral neck in the BW group at week 48 was significantly increased by 8.23% (*P* = 0.031, 95% CI: 0.0039–0.1276). Although the BMD of the other assessed areas in the TC and BW groups tended to increase over time, these differences were not significant. In the control group, the BMD of the lumbar spine (*P* = 0.699, 95% CI: −0.0296–0.0440) and femur tended to decrease over time (femoral neck: *P* = 0.707, 95% CI: −0.0341–0.0501; greater trochanter: *P* = 0.870, 95% CI: −0.0265–0.0313; Ward's triangle: *P* = 0.454, 95% CI: −0.0184–0.0408). Compared with baseline, the BMD of the lumbar spine in the TC group was significantly increased by 5.05% at 4 weeks after exercise cessation (*P* = 0.041, 95% CI: 0.0020–0.0930) and by 5.05% at 8 weeks after exercise cessation (*P* = 0.023, 95% CI: 0.0075–0.0984). Compared with baseline, the BMD of the femoral neck in the BW group was significantly increased by 8.23% at 4 weeks after exercise cessation (*P* = 0.018, 95% CI: 0.0082–0.1318) and by 9.41% at 8 weeks after exercise cessation (*P* = 0.007, 95% CI: 0.0155–0.1391).

The BMD of the lumbar spine was significantly greater in the TC group than the control group at week 48 (*P* = 0.014, 95% CI: 0.0114–0.1286), at 4 weeks after exercise cessation (*P* = 0.023, 95% CI: 0.0071–0.1239), and at 8 weeks after exercise cessation (*P* < 0.001, 95% CI: 0.0384–0.1306). The BMD of the lumbar spine was significantly greater in the BW group than the control group at 8 weeks after exercise cessation (*P* = 0.005, 95% CI: 0.0153–0.1056). The BMD of the femoral neck was significantly greater in the BW group than the control group at week 48 (*P* = 0.009, 95% CI: 0.0144–0.1274), and at 4 weeks after exercise cessation (*P* = 0.001, 95% CI: 0.0308–0.0757) and 8 weeks after exercise cessation (*P* = 0.001, 95% CI: 0.0327–0.1402). At 4 weeks after exercise cessation, the BMD of the femoral neck was significantly greater in the TC group than the control group (*P* = 0.014, 95% CI: 0.0108–0.1223). However, the BMD of the greater trochanter and Ward's triangle did not significantly differ between groups at any timepoint.

## Discussion

The purpose of this study was to compare the effects of 48 weeks of TC and BW exercise on the BMD of perimenopausal women and to analyze the changes in BMD at 4 and 8 weeks after exercise cessation. This information will help clinicians devise effective regular exercise programs to prevent osteoporosis. We found that compared with baseline, the BMD of the lumbar spine (L_2−4_) at week 48 increased by 5.05% in the TC group, and the BMD of the femoral neck at week 48 increased by 8.23% in the BW group. At 4 and 8 weeks after exercise cessation, the BMD of L2-4 in the TC group was still increased by 5.05 and 5.05%, respectively, and the BMD of the femoral neck was still increased by 8.23 and 9.41%, respectively, in the BW group. This study tested the study hypothesis, that regular moderate intensity TC and BW exercises have a positive effect on the BMD of perimenopausal women, and that this effect is maintained after exercise cessation.

After the 48 week TC intervention, the TC group showed a significant 5.05% increase in the BMD of the lumbar spine, and a tendency toward improvement in the femoral BMD. Investigations into the effect of TC exercise on human BMD have focused on the middle-aged and older female population, and the results are controversial. Cheng et al. (11) concluded that 48 weeks of TC (four 70 min sessions/week) significantly improves the BMD of the lumbar spine (L_2−4_) and the femoral neck in older women (mean age 63.3 years); similar results were achieved in older women (mean age 61.3 years) who performed 48 weeks of 30 min TC sessions five times/week (12). Another study found that performing 60 min TC sessions three or six times/week for 48 weeks significantly increases the BMD of the lumbar spine (L_2−4_), femoral neck, and greater trochanter in older women (mean age 64.9 years) (9). In contrast, another study found that 48 weeks of 60 min TC sessions three times/week result in no significant changes in the BMD of the lumbar spine and hip (mean age 69.67 years) (20). The reason for the contrary results of these studies may be related to the different ages of the included subjects, as the effect of exercise intervention on the BMD of humans decreases with aging (4). In addition, the different conclusions among studies may be due to the degree of standardization of TC practice and the individual differences in subjects. When TC exercise was performed, more subjects had less knee flexion, and the strength of the lower limb muscles was reduced with the limitation of the flexion angle (12).

The possible mechanism by which TC improves human BMD was analyzed based on the characteristics of the TC maneuvers. First, the subjects must assume a semi-squatting position for many TC movements, such as “parting the wild horse's mane,” “brush knee and twist step,” and “grasp the bird's tail.” Second, TC exercises emphasize rotation with the waist as the central axis, which requires the participation of both the waist and lower limb muscle groups. In addition, during TC exercise, subjects perform abdominal breathing, which requires more participation of the diaphragm than normal breathing and exercises the muscle groups in the core part of the trunk (21, 22). Therefore, long-term TC exercise produces stress changes in the lumbar spine and femur, which affects the BMD of these sites.

After 48 weeks of BW intervention, the BMD of the femoral neck significantly increased by 8.23% in the BW group, while there were no significant changes in the BMD of the lumbar spine, greater trochanter, and Ward's triangle. The effect of BW exercise on the BMD of older adults is controversial. One study found that performing 60 min sessions of BW exercise five times/week for 10 weeks does not significantly increase the BMD of the upper limbs and calcaneus in perimenopausal women (mean age 50 years) (23). Similarly, another study found that 70 min sessions of BW exercise four times/week for 48 weeks do not cause significant changes in the BMD of older women (mean age 64.9 years) (11). One meta-analysis of studies investigating whether BW exercise improves the BMD of postmenopausal women found that 16 weeks of BW exercise with additional resistance training significantly increases the BMD of Ward's triangle, but not of the lumbar spine and femoral neck (24). However, another meta-analysis of the effect of BW on the BMD of the lumbar vertebrae and femur in perimenopausal and postmenopausal women concluded that BW exercise for different durations (24–96 weeks) does not significantly increase the lumbar BMD, but that BW exercise for longer than 24 weeks significantly increases the BMD of the femoral neck (25). The results of the present study in which perimenopausal women performed 48 weeks of BW intervention were divergent from the abovementioned studies (22, 23), but verified the conclusion of the meta-analysis by Ma et al. (25) that BW exercise significantly increases the BMD of the femoral neck in perimenopausal women. The reason for the contradictory findings among studies may be related to differences in the duration of BW intervention and the exercise frequency. An excessively short intervention time or lower exercise frequency cannot quickly achieve a positive effect. In addition, the contradictory findings may be related to the different ages of the subjects. Compared with previous studies (11, 12, 23, 24), the subjects in the present study were relatively young (mean age 49.7 years). A meta-analysis confirmed our speculation that the effect of exercise on BMD is reduced as the mean subject age increases (4).

The possible mechanism by which BW improves human BMD was analyzed based on the characteristics of BW movement. The frequency of lower limb swing and the force of ground extension are greater during BW exercise than normal walking. Thus, the lower limb muscle groups produce greater stress on the bone during BW compared with normal walking. This may affect the BMD of the proximal femur over the long-term.

The present study showed that BW was superior to TC in increasing the BMD of the femoral neck in perimenopausal women; furthermore, TC tended to be superior to BW in increasing the BMD of the lumbar spine, although this difference was not significant. This suggests that BW may be a better strategy for increasing the BMD of the femoral neck in perimenopausal women, while TC is a better option for increasing the BMD of the lumbar spine. Notably, during the 48-week study period, the BMD of the lumbar spine and femur decreased to varying degrees in the control group, which confirmed that aging has a negative effect on the BMD of perimenopausal women. Thankfully, despite the BMD of the lumbar spine in the TC and BW groups, no significant changes occurred. However, over time, no downward was observed. The present results showed that both regular TC exercise and regular BW exercise alleviate the decrease in BMD caused by aging in perimenopausal women, which is consistent with the conclusion of a previous study that assessed women older than 60 years (11).

The present results showed that the beneficial effect on the BMD of perimenopausal women was maintained after the cessation of TC or BW exercise. At 4 and 8 weeks after exercise cessation, the BMD of the lumbar spine was increased by 5.05 and 5.05% compared with baseline in the TC group, and the BMD of the femoral neck was increased by 8.23 and 9.41% compared with baseline in the BW group. Studies investigating the retainment of the positive effect of exercise on human BMD after exercise cessation have focused on older women. One study in which healthy older women performed 16 weeks of TC and BW intervention and were followed up for 8 weeks found that both TC and BW improve the calcaneal BMD and bone metabolism parameters in older women, and that the BW group had better maintenance of calcaneal BMD improvements than the TC group (16). Another study reported that the BMD of the greater trochanter of the femur in older women who performed 24 weeks of whole-body vibration training was still greater than that in the control group at 8 weeks after exercise cessation (17). The intervention period in the present study was 48 weeks, which expanded the period used in similar previous research. The present results suggest that the beneficial effects on the BMD of perimenopausal women are sustained after the cessation of TC or BW exercise.

The present study has some limitations. First, in the formal trial of this study, 16 samples withdrew because of personal or family reasons, with a sample turnover rate of 17.5% (exceeding the expected value of 15%), involving two less cases than the expected minimum sample. This occurrence may have limited the study's conclusions. Second, considering the limitation of manpower and funds, all the subjects come from the same urban area. Considering the similar living environment, a certain contingency might have been present. Future studies should employ a multicentre RCT trial. Third, we did not measure the biochemical and hormonal indicators related to bone metabolism that affecting BMD and did not increase the physical condition data (e.g., VO_2_ maximal and physiological thresholds of effort). No BMD data were obtained for all subjects at 24 weeks, resulting in unpredictable changes in midtrial data. Fourth, conventional clinical treatment (e.g., estrogen replacement) was not added in this study. Future studies should address these issues individually to effectively explore the effect of TC exercise on BMD in the elderly.

## Conclusion

Forty-eight weeks of moderate intensity TC exercise significantly increased the BMD of the lumbar spine of perimenopausal women, while moderate intensity BW exercise significantly increased the BMD of the femoral neck. These effects were maintained at 4 and 8 weeks after exercise cessation. The present results suggest that both moderate intensity TC and BW reduce the age-related decrease in BMD.

## Data availability statement

The original contributions presented in the study are included in the article/supplementary material, further inquiries can be directed to the corresponding authors.

## Ethics statement

The studies involving human participants were reviewed and approved by the Chengdu Sport University Ethics Committee (approval no. Scsc02). The patients/participants provided their written informed consent to participate in this study.

## Author contributions

LC, SC, BH, and YY contributed to conception, design of the study, and wrote sections of the manuscript. LC and SC organized the database. LC performed the statistical analysis and wrote the first draft of the manuscript. All authors contributed to manuscript revision, read, and approved the submitted version.

## Funding

This study was supported by the Sports Medicine Key Laboratory of Sichuan Province, General Administration of Sport of China (2022-A040), the National Key Research and Development Plan of China (2019YFF0301704), and the project of School of Sports Medicine and Health, Chengdu Sport University (CX21C07).

## Conflict of interest

The authors declare that the research was conducted in the absence of any commercial or financial relationships that could be construed as a potential conflict of interest.

## Publisher's note

All claims expressed in this article are solely those of the authors and do not necessarily represent those of their affiliated organizations, or those of the publisher, the editors and the reviewers. Any product that may be evaluated in this article, or claim that may be made by its manufacturer, is not guaranteed or endorsed by the publisher.
